# CHANGES IN OLDER ADULTS’ INFORMAL CARE NETWORKS

**DOI:** 10.1093/geroni/igz038.3042

**Published:** 2019-11-08

**Authors:** I-Fen Lin, Hsueh-Sheng Wu

**Affiliations:** Bowling Green State University, Bowling Green, Ohio, United States

## Abstract

Many older adults rely on informal care networks to overcome challenges in life and maintain well-being. The composition and function of the informal care network may change as existing caregivers leave and new caregivers join the network over time. The majority of prior studies on caregiving to older adults are based on cross-sectional data and thus cannot examine changes in older adults’ informal care networks. Although some have followed older adults’ informal caregivers over time, they usually focus on primary caregivers, rather than the entire informal care network longitudinally. The newly available panel data on a nationally representative sample of caregivers from the National Study of Caregiving (NSOC) provide an excellent opportunity for researchers to understand how older adults’ informal care networks change over time and what factors relate to discontinuation of care. Using the NSOC 2015 and 2017, we found that 70% of older adults (N = 1,395) experienced changed in informal care networks within two years. Only a small portion of spouses (6%) discontinued giving care to older adults, whereas 21% adult children, 56% other kin, and 77% nonkin stopped caregiving by 2017. We further examined how older adults’ needs for support, caregivers’ resources and constraints, and caregiving experiences were associated with discontinuation of care. This study is expected to advance gerontological research by broadening our understanding of informal caregiving in late life and providing practical implications on how to sustain informal care.

